# Gut flora enhance bacterial clearance in lung through toll-like receptors 4

**DOI:** 10.1186/1423-0127-18-68

**Published:** 2011-09-10

**Authors:** Tzyy-Bin Tsay, Ming-Chieh Yang, Pei-Hsuan Chen, Ching-Mei Hsu, Lee-Wei Chen

**Affiliations:** 1 Department of Surgery, Zuoying Armed Forces General Hospital, Kaohsiung, Taiwan; 2 Department of Surgery, Kaohsiung Veterans General Hospital, Kaohsiung, Taiwan; 3 Department of Biological Sciences, National Sun Yat-Sen University, Kaohsiung, Taiwan; 4 Institute of Emergency and Critical Care Medicine, National Yang-Ming University, Taipei, Taiwan

**Keywords:** gut flora, pneumonia, lipopolysaccharide, Toll-like receptors, NF-κB

## Abstract

**Background:**

The influence of the gut flora on lung inflammatory reaction against bacterial challenge remains undefined. This study was designed to investigate whether gut flora enhances lung defense against *E.coli *pneumonia through TLR4 signaling.

**Methods:**

C3H/HeN (WT) mice and C3H/HeJ (TLR4 deficient) mice were treated with antibiotics in drinking water for 4 weeks to deplete gut commensal microflora. At week 3, drinking water was supplemented with lipopolysaccharide (LPS); a ligand for TLR4, to trigger TLRs in intestinal tract. At the end of 4^th ^week, *E.coli *was injected to trachea to induce *E.coli *pneumonia.

**Results:**

We found that commensal depletion by antibiotic pretreatment before *E.coli *pneumonia challenge induced a 30% decrease of MPO activity in the lung, a significant decrease of bacterial killing activity of alveolar macrophage, and bacterial counts in C3H/HeN mice but not in C3H/HeJ (TLR4 deficient) mice. LPS, a TLR4 ligand, supplementation during antibiotic pretreatment reversed these effects and decreased *E.coli *pneumonia-induced mortality in C3H/HeN mice. Furthermore, commensal depletion induced a suppression of NF-κB DNA binding activity and an increase of KC, MIP-2, IL-1β expression in the lung in C3H/HeN mice but not in C3H/HeJ mice.

**Conclusions:**

Taken together with that commensal depletion increased *E.coli *pneumonia-induced mortality and LPS supplementation decreased it, we conclude that gut flora enhances bacterial clearance against *E.coli *pneumonia through TLR4.

## Background

Lower respiratory infections account for nearly 35% of all deaths from infectious disease. Despite the development of broad-spectrum antibiotics, lower respiratory bacterial infections continue to be a major cause of mortality in both industrialized and developing countries [1,2]. Increased mortality during bacterial pneumonia may have resulted from a failure to control bacterial growth in the lung or to prevent inflammatory injury to the lung. The influence of the gut-lung axis on lung injury and immunity has been known for years, yet the underlying mechanism is not completely understood [3]. It has previously been shown that protecting the integrity of the gut mucosa was effective in reducing idiopathic pneumonia syndrome [3]. Furthermore, clinical trials have demonstrated that enteral feedings significantly reduced the incidence of pneumonia compared to patients fed parenterally [4]. Clearly defining the role of commensal microflora in lung inflammation against pneumonia is warranted to characterize the link between the gut and respiratory tract.

An acute innate immune response in the lung has been characterized by the infiltration of neutrophils [5]. An insufficient neutrophils recruitment leads to life-threatening infection despite the fact that an extreme accumulation of neutrophils results in excessive lung injury association with inflammation. Furthermore, lung content of myeloperoxidase (MPO) is an index to assess the degree of pulmonary neutrophils infiltration. MPO, released by neutrophils, may attack normal tissue and thus contribute to the pathogenesis disease. A better understanding of the mechanisms underlying the regulation of neutrophils influx is crucial to designing improved therapies to augment host defense and attenuate detrimental lung inflammation.

The innate immune system detects the invasion of microorganism through TLRs, which recognize microbial components and trigger inflammatory responses [6]. TLRs are germ line-encoded pattern recognition receptors, and more than 11 members have been identified. Different bacterial products, such as lipopolysaccharide (LPS) and lipoteichoic acid (LTA), were recognized by TLR4 and TLR2, respectively [7]. Previous work has shown that activation of TLRs by LPS administration via the oral route completely protected animals from the dextran sulphate sodium (DSS)-induced inflammatory mortality, morbidity, and severe colonic bleeding seen in mice with depletion of commensal microflora [8]. Also, recent findings suggest that TLR4 plays a critical role in mediating an effective innate immune response against *H. influenzae *in the lung [9]. A major downstream effect of TLR signaling is the activation of the transcription factor NF-κB, which is required for expression of many genes related to innate immunity and inflammation [10]. Previous studies have shown that inflammatory signaling through the NF-κB pathway in airway epithelial cells is critical to regulating the innate immune response against *P. aeruginosa *[11]. Elucidating the key molecules involved with innate pulmonary defense upon recognition of bacteria by pattern recognition receptors are formidable tasks.

Commensal microflora in the intestinal tract could play an important role in the inflammatory reaction of lung against bacterial challenge. The aim of this study is to investigate the role of gut flora in *E.coli *pneumonia-induced lung inflammation. We hypothesized that commensal microflora in the gut could increase lung inflammatory reaction through the toll-like receptors 4 (TLR4). We studied the effect of commensal depletion on *E.coli *pneumonia-induced MPO activity in the lung and the killing activity of alveolar macrophages. Using a commensal depletion model in WT and TLR4 mutant mice, we demonstrate that gut flora are involved in inducing lung inflammatory reaction against bacterial challenge through toll-like receptor 4. Adding TLR ligands in drinking water could be a promising therapeutic strategy to enhance inflammatory reaction against pneumonia in immunocompromised patients.

## Methods

### Animals

Specific pathogen-free male C3H/HeN, weighing between 20 and 25 g were obtained from the National Laboratory Breeding and Research Center (NLBRC, Taipei, Taiwan). C3H/HeJ (TLR4 mutant) mice were purchased from the Jackson Laboratory (Bar Harbor, ME) and bred in the animal room of National Sun Yat-Sen University. C3H/HeJ mice have been demonstrated to have a missense mutation in the third exon of TLR4, yielding a nonfunctional TLR4 [12]. They were fed standard laboratory chow and water *ad libitum *in the animal facility. All animal procedures were in compliance with regulations on animal used for experimental and other scientific purposes approved by the National Sun Yat-Sen University Animal Experiments Committee.

### Depletion of gut commensal microflora and reconstitution of commensal-depleted animals with TLR ligands

Animals are provided ampicillin (A; 1 g/L; Sigma), vancomycin (V; 500 mg/L; Abott Labs), neomycin sulfate (N: 1 g/L; Pharmacia/Upjohn), and metronidazole (M; 1 g/L; Sidmack Labs) in drinking water for four weeks. Previously, a four-week oral administration of vancomycin, neomycin, metronidazole, and ampicillin with the same dose described above in mice has been proved to deplete all detectable commensals [8]. To those animals receiving TLR ligands, drinking water is supplemented with 10 μg/μl of purified *E.coli *026:B6 LPS (Sigma) at week 3 and continued in drinking water for the duration of *E.coli *pneumonia. LPS, a membrane constituent of Gram-negative bacteria, is the best-studied TLR ligand and is recognized by TLR4 and MD-2, a molecule associated with the extracellular domain of TLR4.

### Experimental Design

#### Experiment 1

To examine the role of TLR4 on *E.coli *pneumonia-induced lung inflammatory reaction, C3H/HeN and C3H/HeJ mice were divided into four groups each. Group I (*E.coli *group, n = 6), received *E.coli *intratracheal injection; Group II (LPS + *E.coli *group, n = 6), received LPS supplementation in drinking water for one week and *E.coli *injection; Group III (antibiotic + *E.coli *group, n = 6), received oral antibiotic for four weeks and *E.coli *injection at the end of fourth week. Group IV (antibiotic + LPS + *E.coli *group, n = 6), received oral antibiotic with LPS supplementation and *E.coli *intratracheal injection. At 18 hr after *E.coli *intratracheal injection, animals were sacrificed, lung were harvested for MPO activity assay. At 8 hr after *E.coli *intratracheal injection, lung were harvested for NF-κB DNA-binding activity; IL-1β protein as well as IL-1β, KC, and MIP-2 mRNA expression.

#### Experiment 2

To examine the role of TLR4 in bacterial killing activity of alveolar macrophage, C3H/HeN and C3H/HeJ mice were randomly divided into four groups. Group I (control group, n = 6); Group II (LPS group, n = 6), received LPS supplementation in drinking water for one week; Group III (antibiotic group, n = 6), received oral antibiotic for four weeks; Group IV (antibiotic + LPS group, n = 6), received oral antibiotic with LPS supplementation at week 3. At the end of the 4^th ^week, alveolar macrophages of animals were harvested for the bacterial killing activity assay.

#### Experiment 3

To examine the role of commensal microflora on *E.coli *pneumonia-induced mortality, C3H/HeN were divided into three groups. Group I, received *E.coli *intratracheal injection; Group II, received oral antibiotic for four weeks and *E.coli *intratracheal injection. Group III (antibiotic + LPS + *E.coli *group), received oral antibiotic with LPS supplementation and *E.coli *intratracheal injection. Animals were monitored for mortality after *E.coli *injection for 96 hours.

### Induction of pneumonia

Mice were anesthetized with ketamine hydrochloride (100 mg/kg intramuscularly, Veterinary Laboratories, Wyeth-Ayerst Canada Inc., Mississauga, ON, Canada) and xylazine (5 mg/kg intramuscularly, Bayer Inc., Mississauga, ON, Canada). We have conducted a dose-dependence study with 1.0 × 10^9 ^CFU being the highest dose and found that less dose did not cause pulmonary sepsis and lethality in normal immunocompetent mice. Also, previous paper suggested that intratracheal injection of 1.0 × 10^9 ^CFU *E.coli *could induce significant pneumonia [13]. Therefore, the trachea was surgically exposed and 50 μl (1.0 × 10^9 ^CFU *E.coli*) were instilled via an angiocatheter through the trachea as previous paper suggested [13]. Concentrations of *Escherichia coli *(strain 19138; American Type Culture Collection, Manassas, VA) were determined by colony counting.

### Determination of lung myeloperoxidase activity

Mice were anesthetized and the thorax was opened with median sternotomy. The bilateral lungs and heart were harvested together and the pulmonary vasculature was cleared of blood by gentle injection of 10 ml sterile saline into the right ventricle. The lungs were then blotted dry of surface blood and weighed.

Lung tissues were placed in 50 mM potassium phosphate buffer (pH 6.0) with 0.5% hexadecyltrimethylammonium bromide and homogenized as previously suggested [14]. The homogenate was sonicated on ice and centrifuged for 30 min at 3,000 *g*, 4°C. An aliquot (0.1 ml) of supernatant was added to 2.9 ml of 50 mM potassium phosphate buffer (pH 6.0) containing 0.167 mg/ml of *O*-dianisidine and 0.0005% hydrogen peroxide. The rate of change in absorbance at 460 nm was measured over 3 min. One unit of MPO activity was defined as the amount of enzyme that reduces 1 μmole of peroxide per min and the data were expressed as units per gram of lung tissue (Units/g tissue).

### Processing of lung after exposure to bacteria

Animals were sacrificed by intraperitoneal injection of ketamine (80 mg/kg) and xylazine (10 mg/kg) at 18 hours after *E.coli *intratracheal injection for comparison of their bacterial clearance between different groups of mice. The whole lung was excised and washed with 10 ml of sterile cold saline. The viable bacteria counts of homogenized lung and blood were determined after an 18-hour culture at 37°C in TSB-agar plates. Data were expressed as CFUs per milliliter.

### Western immunoblots

Protein levels of IL-1β in tissue were measured by Western immunoblotting. Tissues were homogenized in protein extract buffer (Sigma) and homogenized samples (50 μg of protein each) were subjected to 12.5% SDS-PAGE under reducing conditions. Proteins were transferred onto PVDF membranes (Millipore) by using a Semi-Dry Electrophoretic system (Bio-Rad). The IL-1β was identified by goat polyclonal antibodies (Santa Cruz Biotechnology Inc., Santa Cruz, CA, USA). The membranes were incubated with the secondary antibodies (Biotinylated anti-rabbit and anti-goat IgG) (Perkin-Elmer Life Science, Boston, USA) for 1 hr at room temperature. Blots were developed by the ECL Western blotting detection reagents (Perkin-Elmer).

### Polymerase chain reaction (PCR) and quantification of PCR products

Total RNA was isolated from cells using TRIZOL reagent (Invitrogen, Life Technologies). Reverse transcription-generated cDNA encoding KC, IL-1β, and MIP-2 genes were amplified using PCR. The sequences are 5' CGTCTAGACTTTCTCCGTTACTTGG3' (antisense) for KC, 5' GAACAAAGGCAAGGCTAACTGA3' (sense) and 5' AACATAACAACATCTGGGCAAT3' (antisense) for MIP-2. 5' CAGCACGAGGCTTTTTTGTTG3' (sense) and 5' TGGTGTGTGACGTTCCCATT3' (antisense) for IL-1β. Meanwhile, we designed one pair of primer: 5' GTGGGCCGCTCTAGGCACCA3' (sense) and 5' CGGTTGGCCTTAGGGTTCAG3' (antisense) for β-actin gene as a control. To a sterile 0.2 ml tube were added 1.5 μl of 10*×Ex Taq*™ buffer, 1.2 μl of dNTP mixture (2.5 mM each), 0.2 μl each of the sense and antisense primers (0.5 mg/ml), 100 ng to 150 ng of the cDNA template and an appropriate amount of water to make a total volume of 15 μl. After adding 0.075 μl of *TaKaRa Ex Taq*™ polymerase (5 units/μl), amplification was performed using a thermocycler (Bio-Rad): 5 min at 95°C before the first cycle, 1 min for denaturation at 95°C, 1 min for annealing at 58°C, and 1 min 30 sec for extension at 72°C, then finally 10 min at 72°C after the last cycle. We recorded the electrophoresis by CCD camera and compared the band intensity by Kodak Digital Science TM ID Image Analysis Software (Eastman Kodak Company).

### Bacterial killing activity of alveolar macrophages

Alveolar macrophages (AM) were harvested from adult C3H/HeN and C3He/HeJ mice by bronchoalveolar lavage (BAL) with Tris-buffered saline containing 0.25 mM EDTA and EGTA. AM were washed three times with RPMI 1640 and counted using trypan blue. AM were collected and resuspended in HBSS as 10^6 ^cells/ml. After 5 min of preincubation, the cell suspension was incubated with *E. coli *(10^8^/ml) at 37°C for 1 h with shaking. The cells were removed as the pellet after centrifugation at 200 *×*g for 10 min, and *E.coli *number in the supernatant was counted [15].

### Statistical Analysis

Values are expressed as means ± standard deviation of the mean, and *p *< 0.05 is considered to be statistical significance. Intergroup comparisons were made using one-way ANOVA followed by Bonferroni correction. Statistical analysis was performed on Prism software (GraphPad). The photographs shown represent the results obtained from at least three independent experiments.

## Results

### Antibiotic pretreatment decreased *E.coli *pneumonia-induced pulmonary neutrophils infiltration and LPS supplementation restored it in C3H/HeN mice but not in C3H/HeJ mice

**Figure 1:**
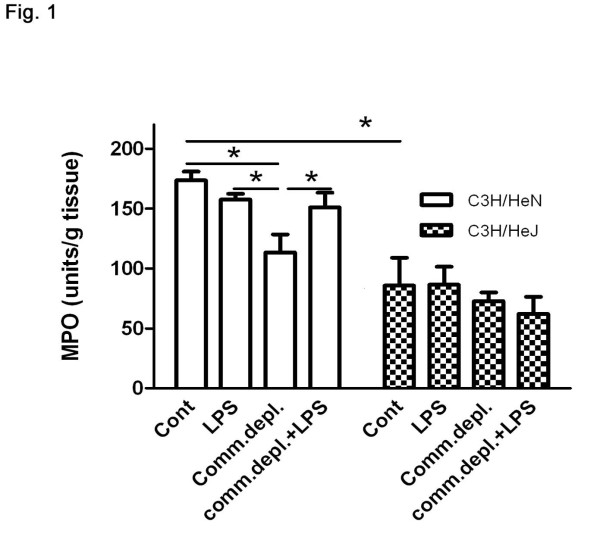
**Commensal depletion decreased *E.coli *pneumonia-induced MPO activity of lung and LPS supplementation reversed it in C3H/HeN mice but not in C3H/HeJ mice (*n *= 6 per group)**. * *p *< 0.05.

To define the role of TLR4 on the inhibitory effect of oral antibiotic pretreatment on *E.coli *pneumonia-induced pulmonary neutrophils infiltration, we examined MPO activity of the lung tissue of C3H/HeJ mice. Intratracheal *E.coli *injection induced a significant three-fold increase of MPO activity in lung compared with that of PBS group (184 ± 21 vs. 39 ± 8 Units/g tissue) in C3H/HeN mice (Figure. 1). LPS treatment with *E.coli *pneumonia did not change MPO activity of lung in comparison with that *E.coli *injection group in C3H/HeN mice. Antibiotic pretreatment with *E.coli *pneumonia significantly decreased 28% of MPO activity in comparison with that of *E.coli *group (127 ± 23 vs. 179 ± 21 Units/g tissue) without antibiotic pretreatment in C3H/HeN mice. LPS supplementation with antibiotic pretreatment significantly increased lung MPO activity by 25% in comparison to that of *E.coli *+ commensal depletion group (149 ± 21 vs. 127 ± 23 Units/g tissue) in C3H/HeN mice (Figure. 1). *E.coli *pneumonia significantly increased MPO activity in the lungs of C3H/HeJ mice compared with that of PBS group (86 ± 18 vs. 31 ± 11 Units/g tissue). Furthermore, C3H/HeJ mice demonstrated a significant 51% decrease of *E.coli *pneumonia-induced lung MPO activity in comparison with that of C3H/HeN mice. Antibiotic pretreatment with or without LPS supplementation did not change *E.coli *pneumonia-induced MPO activity of the lungs in C3H/HeJ mice.

### Antibiotic pretreatment decreased the bacterial killing activity of alveolar macrophages and LPS supplementation restored it in C3H/HeN mice but not in C3H/HeJ mice

**Figure 2:**
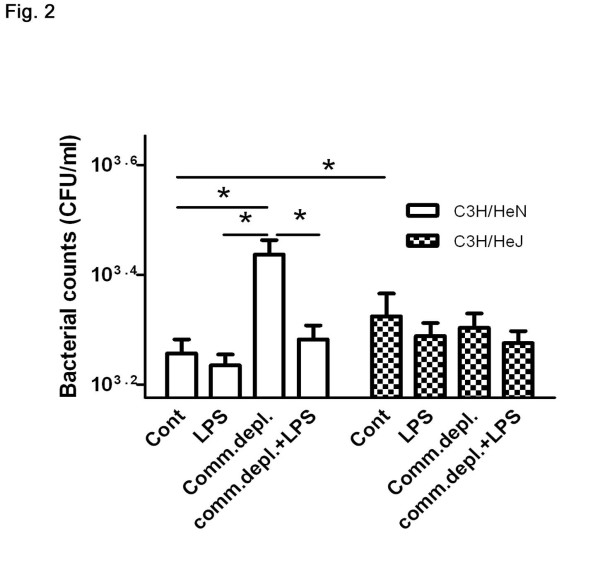
**Commensal depletion (Comm. depl) decreased the bacterial killing activity of alveolar macrophages and LPS supplementation (Comm. depl + LPS) reversed it in C3H/HeN but not in C3H/HeJ mice (*n *= 6 per group)**. * *p *< 0.05.

To further define the effect of oral antibiotic pretreatment on lung defense against pneumonia, we harvested alveolar macrophages from C3H/HeN and C3H/HeJ mice and examined their bacterial killing activity. Alveolar macrophages were harvested and cultured with *E.coli*. Bacterial killing activity of macrophages was determined by counting the *E. coli *number that remained. Antibiotic pretreatment significantly increased bacterial retention by alveolar macrophages compared with that of macrophages from the control group in C3H/HeN mice (2836 ± 370, vs. 1916 ± 250 CFU). LPS supplementation in oral antibiotic significantly decreased 31% bacterial retention by alveolar macrophages compared with that of macrophages from the commensal depletion group in C3H/HeN mice. Furthermore, C3H/HeJ mice demonstrated a significant 22% increase of bacterial retention in comparison with that of C3H/HeN mice. Antibiotic pretreatment with or without LPS supplementation did not change the bacterial killing activity of alveolar macrophages from C3H/HeJ mice (Figure 2). There is no significant difference in alveolar macrophages number harvested between C3H/HeN mice and C3H/HeJ mice (data not show).

### Antibiotic pretreatment enhanced *E.coli *pneumonia-induced bacterial counts in lung in C3H/HeN mice but not in C3H/HeJ mice

**Figure 3:**
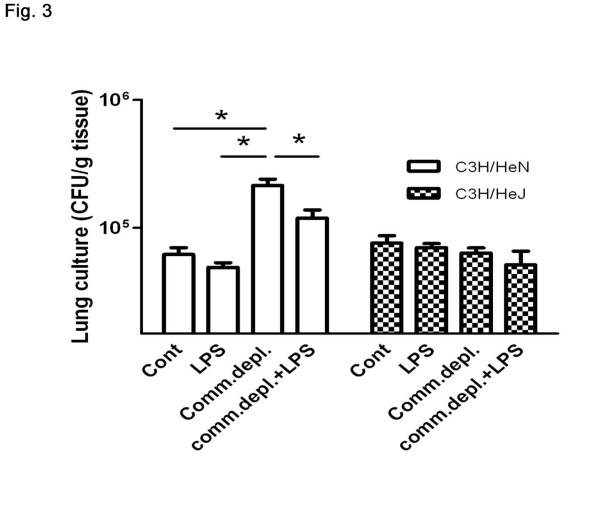
**Antibiotic pretreatment enhanced *E.coli *pneumonia-induced bacterial counts in lung in C3H/HeN mice but not in C3H/HeJ mice**. Antibiotic pretreatment increased *E.coli*-induced bacterial counts in lung and LPS supplementation decreased them in C3H/HeN mice. However, antibiotic pretreatment with or without LPS supplementation did not change *E.coli *pneumonia-induced bacterial counts in lung in C3H/HeJ mice (*n *= 6 per group). * *p *< 0.05.

To further define the role of TLR4 in the effect of commensal microflora on lung immunity, we examined bacterial growth in lung in C3H/HeJ mice after *E.coli *intratracheal injection. Antibiotic pretreatment increased *E.coli*-induced bacterial counts in lung (Figure 3) and LPS supplementation decreased them in C3H/HeN mice. Also, intratracheal injection of *E.coli *(1 ***× ***10^9 ^cfu/mouse) significantly increased lung bacterial counts in C3H/HeJ mice compared with those of PBS injection group. However, antibiotic pretreatment with or without LPS supplementation did not change *E.coli *pneumonia-induced bacterial counts in lung in C3H/HeJ mice.

### Antibiotic pretreatment decreased *E.coli *pneumonia-induced NF-κB activation of lung and LPS supplementation restored it in C3H/HeN mice but not in C3H/HeJ mice

**Figure 4:**
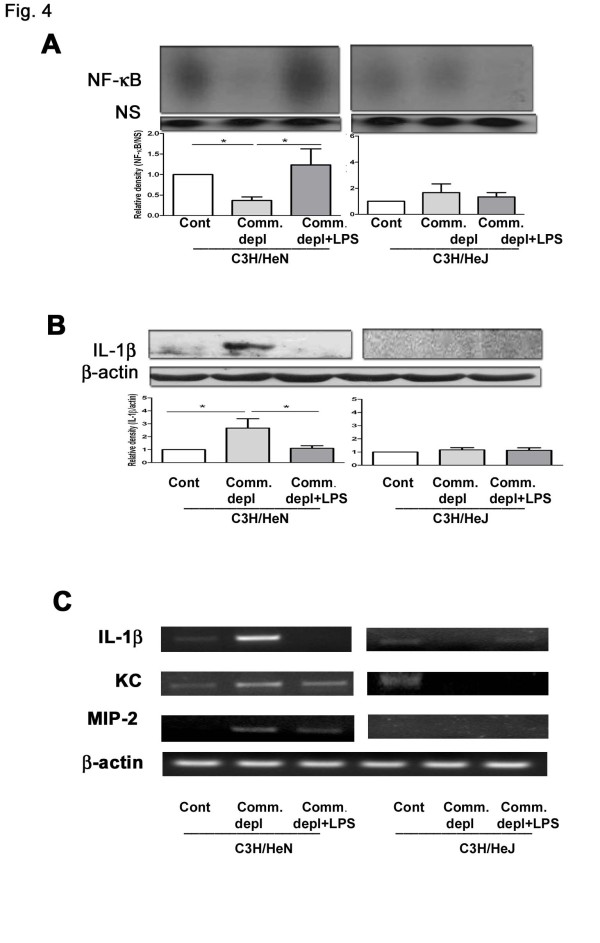
**Commensal depletion decreased NF-κB DNA-binding activity and increased IL-1β, KC, as well as MIP-2 expression in the lung in C3H/HeN mice but not in C3H/HeJ (TLR4 mutant) mice**. (A) Commensal depletion (Comm. depl) decreased the NF-κB activation of lung after *E.coli *pneumonia and LPS supplementation (Comm. depl + LPS) restored it in C3H/HeN mice but not in C3H/HeJ mice (*n *= 5). * *p *< 0.05, vs. control group in C3H/HeN mice. (B) Commensal depletion increased IL-1β protein expression in the lung after *E.coli *pneumonia and LPS supplementation decreased them in C3H/HeN mice but not in C3H/HeJ mice (*n *= 5). Signal intensity was determined by densitometry and normalized to β-actin. * *p *< 0.05, vs. control group in C3H/HeN mice. (C) Commensal depletion increased IL-1β KC, and MIP-2 mRNA expression in the lung after *E.coli *pneumonia and LPS supplementation alleviated them in C3H/HeN mice but not in C3H/HeJ (TLR4-/-) mice. Signal intensity was determined by densitometry and normalized to β-actin. * *p *< 0.05, vs. *E.coli *control group.

To define the role of NF-κB activation in the effect of oral antibiotic pretreatment on lung immunity, we examined the NF-κB DNA binding activity of lung after *E.coli *intratracheal injection. Commensal depletion significantly decreased NF-κB activation of lung after *E.coli *pneumonia in C3H/HeN mice compared with that of control group. LPS supplementation increased the NF-κB activation of lung in C3H/HeN mice compared with that of commensal depletion group (Figure 4). In contrast, antibiotic pretreatment with or without LPS supplementation did not change the NF-κB DNA binding activity of lung in C3H/HeJ mice compared with that of control group (Figure 4A).

### Antibiotic pretreatment increased IL-1β protein as well as IL-1β, KC, and MIP-2 mRNA expression in lung tissue and LPS supplementation reversed them in C3H/HeN mice but not in C3H/HeJ mice

IL-1β expression in lung plays an important role in the inflammatory signaling, and its signaling pathway is critical to the activation of the pro-inflammatory response of inflammatory cells [16]. We examined IL-1β protein and IL-1β, KC, as well as MIP-2 mRNA expression in the lungs in C3H/HeN mice as well as in C3H/HeJ mice. In *E.coli*-treated C3H/HeN mice, antibiotic pretreatment significantly increased IL-1β protein (Figure 4B) and IL-1β, KC, as well as MIP-2 mRNA expression (Figure 4C) in the lung compared with those without pretreatment. LPS supplementation markedly decreased *E.coli *pneumonia-induced IL-1β protein and IL-1β, KC, as well as MIP-2 mRNA expression in the lung compared with those of commensal depletion group in C3H/HeN mice. However, antibiotic pretreatment with or without LPS supplementation did not change IL-1β KC, MIP2 protein and mRNA expression in the lung of C3H/HeJ mice.

### Antibiotic pretreatment enhanced *E.coli *pneumonia-induced mortality and LPS supplementation decreased it in C3H/HeN mice

**Figure 5:**
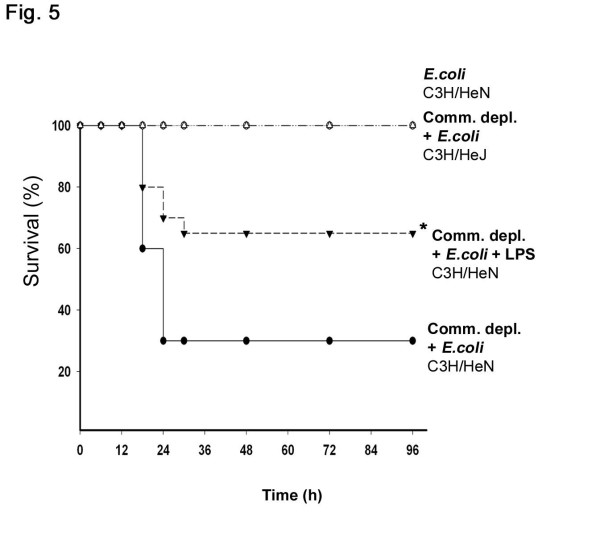
**Antibiotic pretreatment with subsequent *E.coli *intratracheal injection induced an increase of mortality and LPS supplementation decreased it in C3H/HeN mice**. However, in *E.coli*-treated C3H/HeJ mice, antibiotic pretreatment did not induce mortality. * *p *< 0.05, vs. commensal depletion + *E.coli *group (*n *= 15 per group).

To further define the role of gut flora in the *E.coli *pneumonia-induced mortality, we examined the mortality in C3H/HeN mice after antibiotic pretreatment with or without LPS supplementation. *E.coli *intratracheal injection alone did not induce mortality in C3H/HeN mice. Antibiotic pretreatment with subsequent *E.coli *intratracheal injection induced a significant increase of mortality (70%) in C3H/HeN. LPS supplementation with antibiotic pretreatment significantly decreased *E.coli *pneumonia-induced mortality (38%) in C3H/HeN mice (Figure 5). However, in *E.coli*-treated C3H/HeJ mice, antibiotic pretreatment did not induce mortality.

## Discussion

Commensal microflora in the gut are reported to be important regulators for the intestinal haemostasis and the intestinal innate immunity [8]. In the present study, we further demonstrate that gut flora are critical in enhancing lung inflammatory reaction against *E.coli *pneumonia. MPO system plays an important role in the microbicidal activity of neutrophils in the innate immune response to infection [17]. However, an acute innate immune phagocytes response to bacteria in the lung has also been characterized by the infiltration of neutrophils [18], thus, they are necessary for this process. Our data clearly demonstrate that commensal depletion decreases *E.coli *intratracheal injection-induced MPO activity. This indicates that gut flora are important in maintaining neutrophils infiltration in the lung while bacteria invasion. LPS, a TLR4 ligand, supplementation with oral antibiotic pretreatment reverses commensal depletion-induced reduction of MPO activity, suggesting that oral TLR4 stimulation increases neutrophils infiltration in the lung. Moreover, oral antibiotic treatment with or without LPS supplementation does not change *E.coli *pneumonia-induced MPO activity in TLR4 mutant mice. At last, *E.coli *pneumonia induces less MPO activity in TLR4 mutant mice than in WT mice. These suggest that TLR4 signaling pathways are involved in *E.coli *pneumonia-induces neutrophils infiltration in the lung. Altogether, our data demonstrate that gut flora are important in enhancing lung neutrophils infiltration against *E.coli *pneumonia through TLR4 and depletion of TLR4 decreases bacterial challenge-induced lung inflammation.

Next, we try to clarify the effect of commensal depletion on the innate immunity in the lung. Since alveolar macrophages are pivotal to the phagocytic defense in the lung [19], our results indicate that gut commensal microflora are critical in maintaining the bacterial killing activity of alveolar macrophages. First, commensal depletion decreases the bacterial killing activity of alveolar macrophages in WT mice and LPS supplementation reverses its effect. Moreover, alveolar macrophages in TLR4 mutant mice demonstrate a decrease of bacterial killing activity compared with those in WT mice. Also, commensal depletion with or without LPS supplementation does not change the bacterial killing activity of alveolar macrophage in C3H/HeJ mice. This suggests that effect of commensal depletion on the alveolar macrophage is through TLR4. Together, our data indicate that gut flora play an important role in maintaining the bacterial killing activity of alveolar macrophages through TLR4 and depletion of TLR4 decreases the bacterial killing activity of alveolar macrophages.

NF-κB family members control transcriptional activity of various promoters of proinflammatory cytokines, cell surface receptors, transcription factors, and adhesion molecules that are involved in inflammation such as TNFα, ICAM, KC, and MIP-2 [20]. Previous studies have shown that TLR4 stimulation could maintain intestinal haemostasis through the NF-κB activation of the intestinal mucosa [8]. NF-κB activation is an essential immediate early step in innate immune cells activation [21]. The inhibitory effect of oral antibiotic pretreatment on NF-κB DNA binding activity in the lung further corroborates the important role that commensal microflora play in inducing NF-κB signaling in the lung. Nuclear factor kappa B (NF-κB) regulates the transcription of a wide array of gene products that are involved in the molecular pathobiology of the lung [22]. Three lung cell types, epithelial cells, macrophages and neutrophils, have been shown to be involved in the generation of lung inflammation through signaling mechanisms that are dependent on activation of the NF-κB pathway [22]. Inflammatory signaling through the NF-κB pathway by airway epithelial cells critically regulates the innate immune response to *P. aeruginosa *[11]. Our present results further suggest that commensal microflora in intestinal tract are critical in inducing the NF-κB activation and lung defense against *E.coli *pneumonia. Moreover, the abolition of the stimulatory effect of LPS on pulmonary NF-κB activation and bacterial killing activity of alveolar macrophages in TLR4 mutant mice further corroborates that gut flora are important in enhancing NF-κB activation in the lung through TLR4 and LPS supplementation enhances lung defense through the TLR4 and NF-κB signaling pathways.

Both polymicrobial sepsis and intratracheally lipopolysaccharide (LPS) injection can induce acute lung inflammation with elevated IL-1β, KC, MIP-2 levels and MPO activity of lung in mice [23]. Interleukin-1β (IL-1β) has been shown to induce the expression of intercellular adhesion molecule-1 (ICAM-1) on airway epithelial cells and contributes to inflammatory responses [24]. Our data demonstrate that commensal depletion decreases MPO activity and NF-κB activation but induces IL-1β, KC, and MIP-2 expression of lung after *E.coli *pneumonia. Previously, mice deficient in TLR4 demonstrated a substantial delay in clearance of *H. influenzae *with diminished IL-1β, IL-6, TNFα, MIP-α, and MIP-2 in bronchoalveolar lavage [9]. Similarly, our present data demonstrate that oral antibiotic pretreatment with *E.coli *pneumonia-induced NF-κB activation as well as IL-1β, KC, and MIP-2 expression of lung are decreased in C3H/HeJ mice. Altogether, our data suggest that commensal microflora are critical in decreasing KC, MIP-2, and IL-1β of lung in response to *E.coli *pneumonia.

More importantly, commensal depletion increases *E.coli *pneumonia-induced mortality and LPS supplementation significantly decreases it in WT mice. This further corroborates that gut commensal microflora is critical in maintaining lung defense against bacterial challenge through the increase of the bacterial killing activity of alveolar macrophage and neutrophils infiltration. Our recent work have demonstrated that commensal gut depletion by antibiotic pretreatment before *E.coli *pneumonia challenge in WT mice induced a 15-fold and a 3-fold increase in bacterial counts in blood and lung, respectively, and a 30% increase of mortality when compared with the *E.coli *group [25]. Our present data further demonstrate that *E.coli *pneumonia challenge induced a 30% decrease of MPO activity in the lung and a significant decrease of bacterial killing activity of alveolar macrophage in WT mice but not in TLR4 deficient mice. Altogether, our data indicate that commensal flora play an important role in maintaining lung inflammation reaction against *E.coli *pneumonia through TLR4. Our data also imply that early enteral feeding to restore commensal microflora or adding TLRs ligands in diet might be a feasible way to increase host defense against pneumonia in intensive care patients.

## Conclusions

From our present results, the mechanism by which commensal microflora regulate *E.coli *pneumonia-induced lung inflammatory reaction has been described. Commensal microflora increase NF-κB DNA binding activity but decrease IL-1β, KC, as well as MIP-2 expression in the lung after *E.coli *pneumonia. Commensal microflora also enhance neutrophils infiltration and the killing activity of alveolar macrophage against *E.coli *pneumonia through TLR4.

## Competing interests

The authors declare that they have no competing interests.

## Authors' contributions

LWC and CMH designed research; TBT and PHC performed research; LWC and CMH analyzed data; LWC and CMH wrote the paper. All authors read and approved the final manuscript.
